# The Reuse of Healing Abutments: A Questionnaire-Based Survey

**DOI:** 10.7759/cureus.69054

**Published:** 2024-09-10

**Authors:** Anup Shelke, Surabhi Tandel, Chitrika Subhadarsanee, Subodh Gaikwad, Ranu Oza

**Affiliations:** 1 Department of Periodontology, Dr. Hedgewar Smruti Rugna Seva Mandals Dental College and Hospital, Hingoli, IND; 2 Department of Periodontology, Sharad Pawar Dental College and Hospital, Datta Meghe Institute of Higher Education and Research, Wardha, IND

**Keywords:** dental implants, disinfection, healing abutment, reuse, sterilization

## Abstract

Healing abutments (HA) are used in second-stage implant surgery to create the optimal peri-implant environment. Despite being a component intended only for short-term use, it remains in the mouth for a few weeks to several months and stays in close contact with organic debris, intraoral fluids, and bacteria. Reusing the HAs modifies their surfaces, expands the coating layer's porosity, and promotes bacterial colonization at the HA-implant interface in addition to the previous contamination. Therefore, the study's goals were to determine the frequency of HA reuse, examine the sterilizing and disinfection techniques employed, and examine the factors that contributed to the dental professionals in the Indian state of Maharashtra utilizing these components again when placing dental implants. For the study, 150 dentists who worked in the field of implantology were chosen. An online questionnaire issued via email and WhatsApp was used to collect data, which started in January 2023. The survey was designed to store the demographic information and responses of the participants, and it was made available through Google Forms. By the end of February 2023, the responses had been gathered. Tables and graphs were used to present the study outcomes that were based on the statistical analysis. The analysis employed the Chi-square test, with a p-value of 0.05 deemed statistically significant. There were 150 answers in all, with 44% women and 56% men. Merely 7.4% of participants do not reuse healed abutments, compared to 92.6% who follow this procedure. The respondents were also asked about the number of times they reused the HAs, 69% of the implantologists reused the same component countless times, while 31% reused it only once. All the respondents reported sterilizing the HAs before reuse and the method used for sterilization was autoclave (96.4%) and UV chamber (3.6%). In response to a question concerning informing patients about the reuse of HAs, 77.6% of implantologists stated they do not interact with patients, while only 22.4 reported doing so. Implantologists in the state of Maharashtra, India reuse HAs and use heterogeneous methods for disinfection and autoclave sterilization.

## Introduction

Healing abutments (HAs) are used in second-stage implant surgery to create the optimal peri-implant environment. Its contribution to intensifying aesthetics has been better appreciated in the past decade. HA allows the maturation of gingival epithelium around the implant and controls the unforeseeable tissue shrinkage before a definitive prosthesis has been delivered [1]. Despite being a component intended only for short-term use, it remains in the mouth for a few weeks to several months and stays in close contact with organic debris, intraoral fluids, and bacteria [2]. These elements are exposed to create a biofilm that is firmly adherent and home to many bacterial and fungus species [3].

By definition, HAs are "indwelling," or in-situ intermediate, devices that dentists have historically reused because of financial constraints [4]. HA is contaminated by a multitude of substances, including blood, saliva, food particles, and epithelial cells [5]. The mechanical and biological factors that determine the HA in situ are influenced by the oral microbiota, smoking status, nutrition, and dental hygiene practices of each patient. A few authors have discussed the biological and mechanical issues surrounding the reuse of HAs, such as the incapacity to thoroughly remove contaminants from the HAs prior to reusing and the device's inherent loss of functionality after several uses [4-7].

Since titanium makes up the majority of abutments, it has been assumed that autoclave sterilization ensures the security of this type of reuse [8]. Reusing the HAs modifies their surfaces, expands the coating layer's porosity, and promotes bacterial colonization at the HA-implant interface in addition to the previous contamination. This may also cause the peri-implant tissues to become chronically inflamed [2]. Regarding patient safety, ethics, and financial concerns, Cakan et al. observed that there is disagreement about the re-sterilization of implant components [4]. Dental practitioners who reuse HA contend that the hazards outweigh the advantages, whereas manufacturers designate these devices for single use in order to preserve their profit margin [4].

Owing to the increased likelihood of sterilization and disinfection process failure, as well as the ongoing danger of bacterial contamination and HA structural alterations, experts need to be knowledgeable and informed of how to handle these components. In addition, it is crucial to comprehend their awareness of the constraints and dangers associated with reusing these HAs. Therefore, the study's goals were to determine the frequency of HA reuse, examine the sterilizing and disinfection techniques employed, and examine the factors that contributed to the dental professionals in the Indian state of Maharashtra utilizing these components again when placing dental implants.

## Materials and methods

The sample size was calculated using N=(Z_α/2_)^2^s^2^/d^2^ where s=standard deviation, d= accuracy of estimate, (Z_α/2_)= normal deviation. G-Power software version 3.1.9 (Heinrich Heine University Düsseldorf, Düsseldorf, Germany) was used to calculate the sample size for this study at a 95% confidence interval and 95% power of the study. For the study, 150 dentists were chosen. Both male and female dental practitioners practicing implantology aged between 25 and 65 years. Dental Practitioners with BDS, postgraduate students, MDS, PHD, diploma, fellowship in oral implantology degree. Practitioners have their own clinical setup or consulting practitioners. Practitioners are practicing in rural or urban areas. Those practitioners who disagreed to participate in the study were excluded. For participants who agreed to participate in the study, informed consent was obtained. Ethical approval was taken from the institute's ethical committee. An online questionnaire issued via email and WhatsApp was used to collect data, which started in January 2023. After seven days, the dental professionals who failed to reply to the questionnaire again got it, for a maximum of two submissions. The survey was designed to store the demographic information and responses of the participants, and it was made available through Google Forms. The survey was composed of two sections: a first section with seven questions about demographics, and a second section with ten questions about reuse, disinfection and sterilization techniques, manufacturer guidelines, restrictions on reusing, and patient communication regarding reusing the component. By the end of February 2023, the responses had been gathered. Version 22 of the Statistical Package for Social Sciences (SPSS) (IBM Corp., Armonk, NY, USA) was used to perform the study's statistics. After being coded, the gathered data was imported into SPSS for statistical analysis. Mean and standard deviation were used to characterize quantitative data, whereas frequency and percentage were used to characterize qualitative data. Tables and graphs were used to present the study outcomes that were based on the statistical analysis. The analysis employed the Chi-square test, with a p-value of 0.05 deemed statistically significant.

## Results

**Figure 1:**
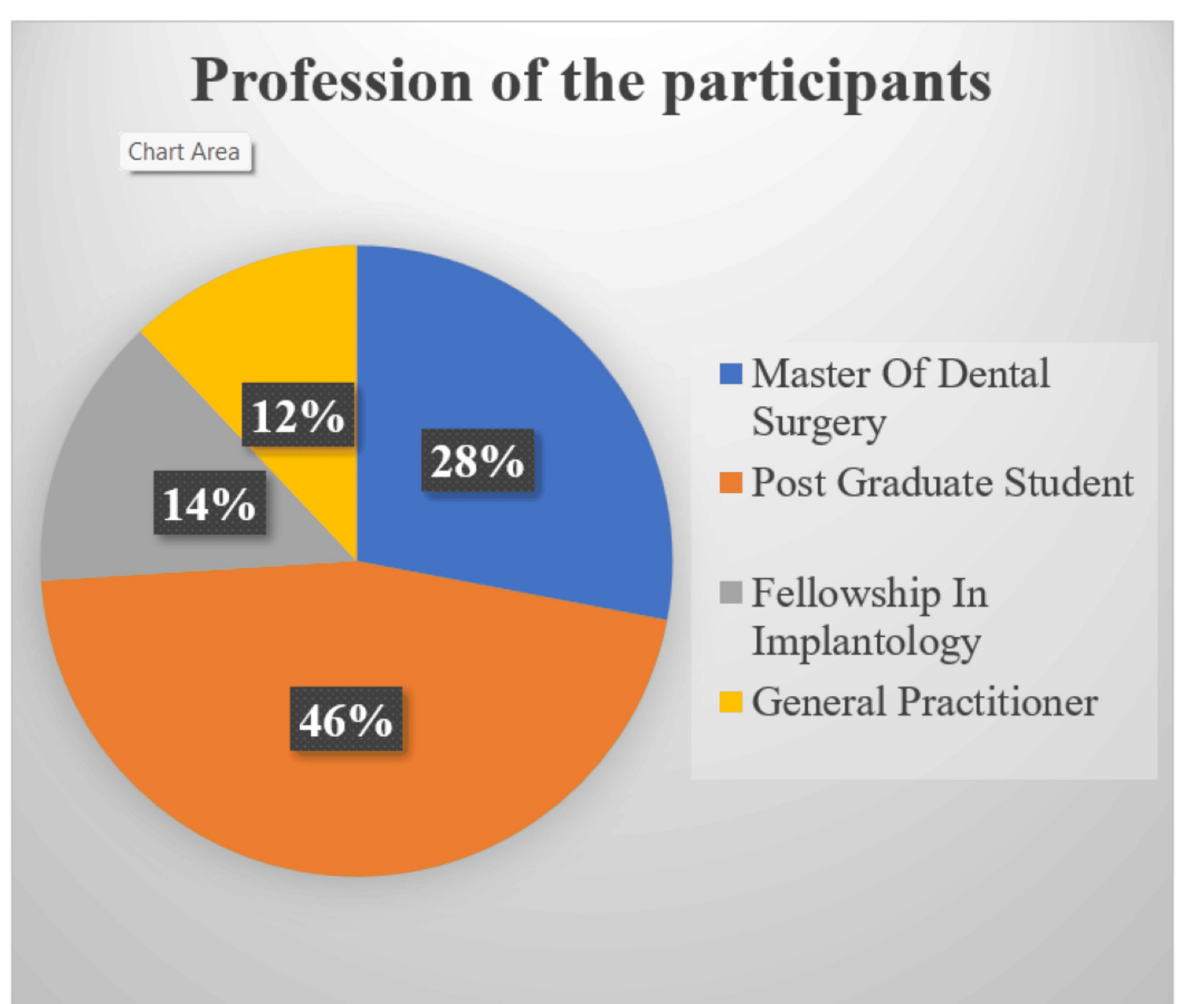
Profession of the participants

There were 150 answers in all, with 44% (n=66) women and 56% (n=84) men. With a standard deviation of 6.2 years, the average age was 35.3 years. The age range of the majority of responders (35.5%) was between 30 and 40. Out of 150 participants who practiced implantology; 46% (n=69) had done specialization in the field of implantology, 28% (n=42) were pursuing specialization, 14% (n=21) had persuaded fellowship in implantology, and 12% (n=18) were general practitioners (Figure 1).

**Figure 2:**
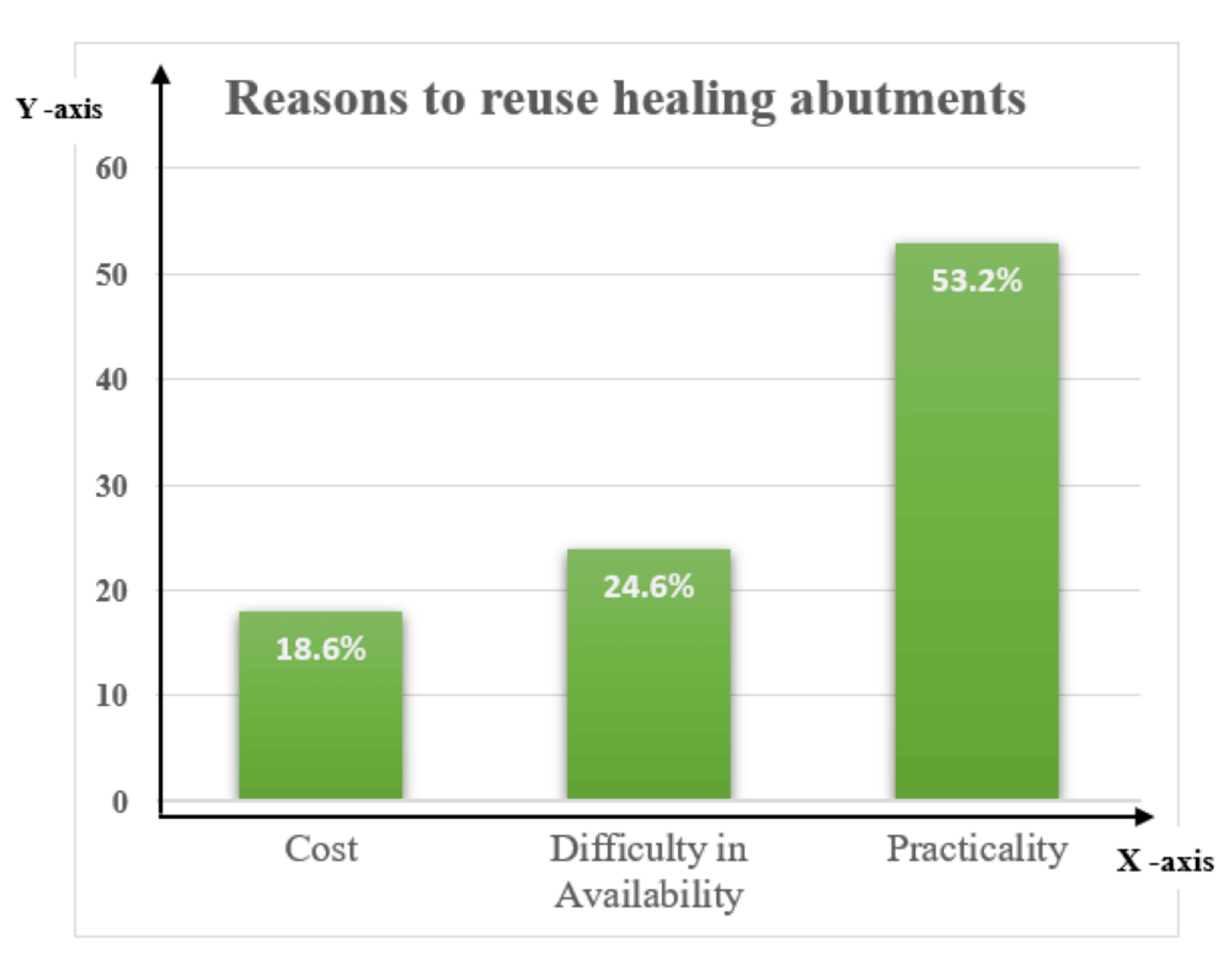
X-axis: reasons to reuse HAs; Y-axis: percentage of practitioners reusing HAs HA: healing abutment

Merely 7.4% (n=11) of participants do not reuse healed abutments, compared to 92.6% (n=139) who follow this procedure. When asked what the primary motivation was for reuse, 53.2% (n=74) said it had to do with practicality, while 24.6% (n=34) said it had to do with availability issues. Cost was the response from 18.6% (n=31) (Figure 2).

**Figure 3:**
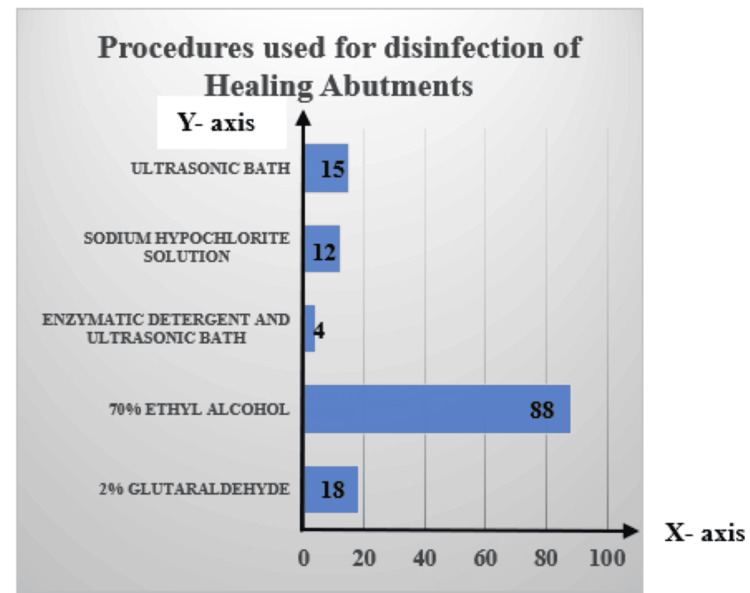
X-axis: percentage of implantologist disinfecting HAs; Y-axis: different methods of disinfection used HA: healing abutment

The respondents were also asked about the number of times of reused the HAs, 69% (n=96) of the implantologists reused the same component countless times, while 31% (n=43) reused it only once. Of the 139 participants who repurposed the HAs, 98.5% (n=137) reported that they cleaned the abutments after each use. The methods most frequently employed to disinfect the components were as follows: 70% used ethyl alcohol (64.2%, n=88), 13.1% used glutaraldehyde (2%, n=18), 10.9% used an ultrasonic bath (n=15), 8.8% used sodium hypochlorite solution (n=12), and 3% used enzymatic detergent with an ultrasonic bath (n=4) (Figure 3).

All the respondents reported sterilizing the HAs before reuse and the method used for sterilization was autoclave 96.4% (n=134) and UV chamber 3.6% (n=5). When asked whether the manufacturers had given them any instructions on how to reuse the HAs, 71.2% (n=99)of them replied that they had not. When questioned about their knowledge of the restrictions pertaining to the reutilization of HAs, 68.3% (n=95) of implantologists said they were not aware of them, while 31.7% (n=44) did. In response to a question concerning informing patients about the reuse of HAs, 77.6% (n=108) of implantologists stated they do not interact with patients, while only 22.4% (n=31) reported doing so.

## Discussion

In the Indian state of Maharashtra, 92.6% of implantologists employ the reuse of HAs. The results found authentication from the information reported by several authors [4,9-11]. It was discovered that professionals primarily reused HAs for practical reasons. However, the rationale pragmatism is very debatable in terms of patient safety and ethics, which clarifies why 77.6% of implantologists did not let patients know that the component may be reused [9]. Several disinfection procedures were reported, out of which 64.2% of the implantologists used 70% ethyl alcohol as a disinfectant for removing the organic matter. However, according to a systematic review by Kyaw et al. wiping the abutment surface with alcohol has the lowest decontamination efficacy [12]. 13.1 % of respondents responded that they used 2% glutaraldehyde and 10.9% used an ultrasonic bath to remove the organic matter deposited on the components.

In a study comparing the disinfection effectiveness of 99.9% ethyl alcohol, 6% hydrogen peroxide (H_2_O_2_), and 2% glutaraldehyde on contaminated diagnostic instruments, Ganavadiya R et al. (2014) discovered a considerable decrease in the total viable count with all of the disinfectants [13]. After disinfection, none of the disinfectants were able to totally eradicate the microbiological contamination, nevertheless. Even the effectiveness of removing the organic matter from the HAs using an ultrasonic bath is extremely low [5]. 96.4% of the implantologists used an autoclave, and 3.6% used a UV chamber as a sterilization mode. On the other hand, the HAs experience surface alterations like porosities from the oxidation of the titanium coating layer [2]. Due to bacterial adherence on the surface of HAs, these porosities serve as a supporting element in the formation of a complex biofilm that houses a variety of pathogenic species and ultimately causes inflammation of the tissues around the implant [3].

Maintenance of oral hygiene by the patient and anatomical location of HAs in the oral cavity may also contribute to the retention of biofilm. The presence of biofilm on the HAs presents an alarming sign of the occurrence of peri-implantitis in proper use in the future. This risk was perceived by only 31.7% of the individuals, while 68.3% of implantologists reported that they were unaware of the limitations. This response strongly correlates with the insufficient availability of information on the proper use of HA, as 71.2% of the respondents reported that they did not receive any guidelines from the manufacturers who are involved in manufacturing the components. Numerous investigations have revealed that there is an elevated risk of HA contamination even during sterilization and disinfection [5,6,14,15]. The current study's findings can aid in recognizing the restrictions and health hazards related to reusing these components.

Limitation

The small sample size has limited the statistical analysis of the results. Hence, a larger sample size would be required. The study should be conducted on general dental practitioners, implantologists, and postgraduate students individually so that the selection bias would have been eliminated.

## Conclusions

Implantologists in the state of Maharashtra, India reuse HAs and use heterogeneous methods for disinfection and autoclave sterilization. The unperceiving blindness about the risks associated with the reuse of the HAs and the lack of guidelines from the manufacturer companies prevailed amongst the implantologists participating in the study.

## Appendices

Questionnaire:

1.     Do you reuse healing abutments?

a)     Yes

b)     No

2.     If yes, then why do you reuse it?

a)     Cost

b)     Difficulty in availability

c)     Practicality

d)     Any other reason____________________________________________________

3.     How many times do you reuse the same healing abutment?

a)     Only once

b)     Countless number of times

4.     Do you disinfect the healing abutment after use?

a)     Yes

b)     No

5.     If yes, then which procedure do you follow for disinfection?

a)     Ultrasonic bath

b)     Enzymatic detergent and ultrasonic bath

c)     Sodium bicarbonate

d)     Sodium hypochlorite solution

e)     2% glutaraldehyde

f)      70% ethyl alcohol

6.     Do you sterilize the healing abutment before reusing?

a)     Yes

b)     No

7.     If yes, then which sterilization protocol do you follow?

a)     Autoclave

b)     Hot air oven

c)     UV chamber

d)     Ethylene oxide gas (EtO)

8.     Do you receive any guidelines from the manufacturers on reusing the healing abutment?

a)     Yes

b)     No

9.     Are you aware of the limitations associated with the reuse of healing abutments?

a)     Yes

b)     No

10.  Do you communicate with the patient about reusing the component?

a)     Yes

b)     No
